# Prevention of zoonotic spillover: From relying on response to reducing the risk at source

**DOI:** 10.1371/journal.ppat.1011504

**Published:** 2023-10-05

**Authors:** Authored by the members of the One Health High-Level Expert Panel (OHHLEP), Wanda Markotter, Thomas C. Mettenleiter, Wiku B. Adisasmito, Salama Almuhairi, Casey Barton Behravesh, Pépé Bilivogui, Salome A. Bukachi, Natalia Casas, Natalia Cediel Becerra, Dominique F. Charron, Abhishek Chaudhary, Janice R. Ciacci Zanella, Andrew A. Cunningham, Osman Dar, Nitish Debnath, Baptiste Dungu, Elmoubasher Farag, George F. Gao, David T. S. Hayman, Margaret Khaitsa, Marion P. G. Koopmans, Catherine Machalaba, John S. Mackenzie, Serge Morand, Vyacheslav Smolenskiy, Lei Zhou

**Affiliations:** 1 Centre for Viral Zoonoses, Department of Medical Virology, Faculty of Health Sciences, University of Pretoria, Pretoria, South Africa; 2 Friedrich-Loeffler-Institut, Federal Research Institute for Animal Health, Greifswald-Insel Riems, Germany; 3 Universitas Indonesia, Depok, West Java, Indonesia; 4 National Emergency Crisis and Disasters Management Authority, Abu Dhabi, United Arab Emirates; 5 Centers for Disease Control and Prevention, Atlanta, Georgia, United States of America; 6 World Health Organization, Guinea Country Office, Conakry, Guinea; 7 Institute of Anthropology, Gender and African Studies, University of Nairobi, Nairobi, Kenya; 8 National Ministry of Health, Autonomous City of Buenos Aires, Argentina; 9 School of Agricultural Sciences, Universidad de La Salle, Bogotá, Colombia; 10 Visiting Professor, One Health Institute, University of Guelph, Guelph, Ontario, Canada; 11 Department of Civil Engineering, Indian Institute of Technology (IIT), Kanpur, India; 12 Brazilian Agricultural Research Corporation (Embrapa), Embrapa Swine and Poultry, Concórdia/SC, Brazil; 13 Institute of Zoology, Zoological Society of London, London, United Kingdom; 14 Global Operations Division, United Kingdom Health Security Agency, London, United Kingdom; 15 Global Health Programme, Chatham House, Royal Institute of International Affairs, London, United Kingdom; 16 Fleming Fund Country Grant to Bangladesh, DAI Global, Dhaka, Bangladesh; 17 Onderstepoort Biological Products SOC (OBP), Afrivet, B M, Pretoria, South Africa; 18 Faculty of Veterinary Science, University of Kinshasa, Kinshasa, DR Congo; 19 Ministry of Public Health, Health Protection & Communicable Diseases Divison, Doha, Qatar; 20 Chinese Center for Disease Control and Prevention, Beijing, P.R. China; 21 Molecular Epidemiology and Public Health Laboratory, Massey University, Palmerston North, New Zealand; 22 Mississippi State University, Starkville, Mississippi, United States of America; 23 Erasmus MC, Department of Viroscience, Rotterdam, the Netherlands; 24 EcoHealth Alliance, New York, New York, United States of America; 25 Faculty of Health Sciences, Curtin University, Perth, Australia; 26 IRL HealthDEEP, CNRS - Kasetsart University - Mahidol University, Bangkok, Thailand; 27 Federal Service for Surveillance on Consumer Rights Protection and Human Well-being (Rospotrebnadzor), Moscow, Russian Federation; Boston Children’s Hospital, UNITED STATES

## Background and context

The devastating impact of Coronavirus Disease 2019 (COVID-19) on human health globally has prompted extensive discussions on how to better prepare for and safeguard against the next pandemic. Zoonotic spillover of pathogens from animals to humans is recognized as the predominant cause of emerging infectious diseases and as the primary cause of recent pandemics [1]. This spillover risk is increased by a range of factors (called drivers) that impact the nature, frequency, and intensity of contact between humans and wild animals. Many of these drivers are related to human impact, for example, deforestation and changes in land use and agricultural practices. While it is clear that the triad of prevention-preparedness-response (P-P-R) is highly relevant, there is much discussion on which of these 3 strategic activities in the field of emerging infectious disease should be prioritized and how to optimally target resources. For this, it is important to understand the scope of the respective activity and the consequences of prioritization.

Already, the World Bank Pandemic Fund and forthcoming global Pandemic instrument [2] negotiated by the World Health Organization (WHO) [3] appear primarily focused on the early detection, and reaction to the appearance of human illnesses, often with explicit focus only on action to be taken once pathogen spillover and spread have occurred. Strategies to reduce the probability of spillover events are under-prioritized and underutilized, as highlighted by recent infectious disease crises such as Ebola and Mpox epidemics, and have been lost in overall preparedness discussions and recovery financing. This “more of the same” focus suggests that it is politically more expedient to allocate financial resources to deal with a problem once it has arisen, rather than taking the steps necessary to reduce the risk of it occurring in the first place. It is often claimed that allocating resources to prevent something from happening is politically difficult as the value of prevention is largely “invisible” (prevention paradox) or it will take a long time to show effects. However, there are now several communications highlighting the economic benefits of prevention of spillover [1,4,5]. If taken, actions to prevent spillover are estimated at $10 to 31 billion per year globally, as a cumulative investment from preventive actions achievable by specific industries. However, addressing the drivers of pathogen spillover through a One Health approach has significant subsequent economic co-benefits; for example, reducing deforestation is estimated to create $4 billion per year in social benefits from reduced greenhouse gas emissions [4]. COVID-19 has demonstrated the immense burden of a pandemic, including significant mortality resulting in economic recession, with the global economy contracting by 4.4 percent in 2020. The expected economic losses from this pandemic are estimated at nearly $14 trillion up to 2024 [6,7]. These losses parallel those incurred by other infectious disease emergencies, including the 2003 SARS pandemic with an estimated economic loss of $52 billion; the Ebola virus disease outbreak in West Africa in 2014 to 2016 with a GDP loss of $2.8 to 32.6 billion and the comprehensive economic and social burden estimated to be $53.19 billion [8]; and the 2015 to 2016 Zika virus disease outbreak with an estimated loss in the United States, Caribbean, and Latin America of $20 billion [9,10]. If invested in, prevention strategies would reduce the likelihood of another pandemic substantially and likely generate sufficient return on investment over time while also having the potential to generate substantial co-benefits [1,10]. Prevention is already valued in other sectors: policymakers and industries have led on prevention in other areas, such as expenditure on counter-terrorism, driving laws and insurance incentives to reduce the frequency of traffic accidents, on the nuclear deterrent, and in some cases on flood prevention and other water management measures, exemplifying a political willingness to spend vast sums of money to preempt a harmful event in certain areas or circumstances, but not on pandemic prevention.

## Defining “prevention of spillover’

It is essential to define “prevention of spillover” in the context of preventing outbreaks, endemicity of diseases, epidemics, and pandemics to ensure alignment with prioritization of actions and resources. At present, the term “prevention” is used differently in different contexts. For example, in public health it refers to prevention of human disease from occurring at all (primary prevention) or prevention of small localized disease outbreaks in people from spreading and developing into an epidemic or pandemic (downstream/secondary prevention). Secondary prevention is often achieved by interventions such as early detection, vaccines, improved health systems, drug therapy, health promotion and social and behavior change, and implementation of sanitary measures. Secondary prevention could be better referred to as “containment of infection,” as this clearly describes the objective of these measures while avoiding potential confusion with prevention of spillover.

Prevention of spillover in the context of this paper refers to preventing the critical first step, i.e., preventing a pathogen from transferring from animals to humans. While this paper specifically addresses pandemic prevention in humans, in line with the OHHLEP One Health definition endorsed by the Quadripartite, it is important to note that pathogen spillover from humans to other species or between other species facilitated by human activity (e.g., wildlife trade) can also have devastating impacts on wild and domestic animal populations.

**Fig 1:**
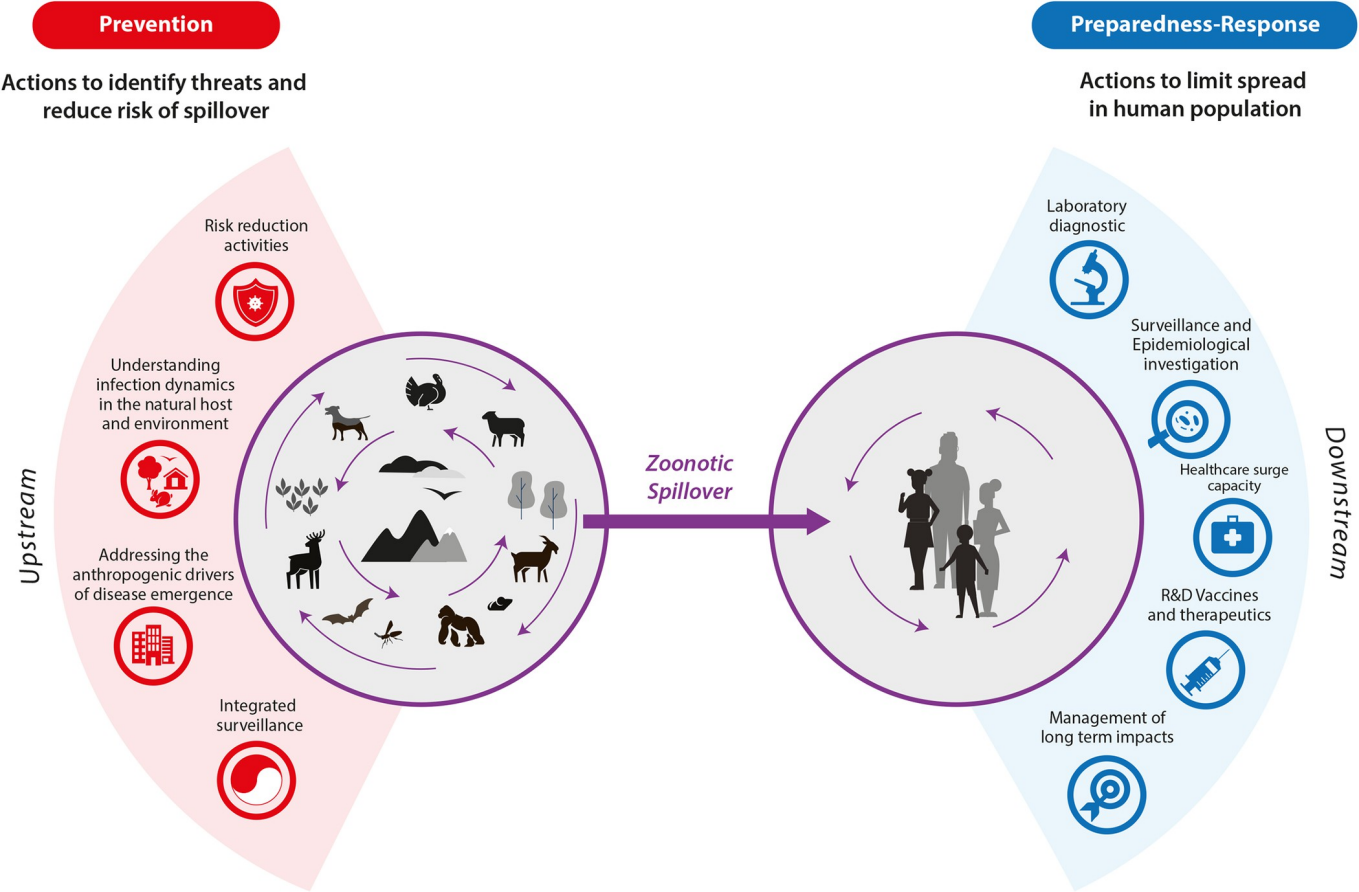
Prevention of zoonotic spillover to humans: Prevention of pathogen spillover from animals to humans; shifting the infectious disease control paradigm from reactive to proactive (primary prevention, upstream). Prevention includes addressing the drivers of disease emergence, namely ecological, meteorological, and anthropogenic factors and activities that increase spillover risk, in order to reduce the risk of human infection. It is informed by, among other actions, biosurveillance in domestic and wild animals, people and the environment, understanding pathogen infection dynamics, and implementing intervention activities. Exemplary preparedness-response actions (downstream actions) are indicated on the right.

Prevention of spillover can be enacted by addressing drivers of pathogen spillover in a One Health approach at the human–animal–environment interface to minimize the risk of human infection by zoonotic pathogens, including interventions such as vaccines. To be clear on the prioritization of preventing future epidemics that can lead to pandemics, we propose a definition of prevention that focuses on the prevention of zoonotic spillover, i.e., all upstream events that have an impact on pathogen spillover (**Box 1**, **Fig 1**, and **Table 1**), whereas downstream activities are contained within the preparedness and response actions. Prevention of pathogen spread in humans (secondary prevention) specifically involves containment measures that need to be in place after spillover of pathogens to the human population—these measures may be implemented both in the public health sector, in the animal health sector, and in the environment.

**Box 1. Prevention of zoonotic spillover to humans**

Prevention of pathogen spillover from animals to humans; shifting the infectious disease control paradigm from reactive to proactive (primary prevention). Prevention includes addressing the drivers of disease emergence, namely ecological, meteorological, and anthropogenic factors and activities that increase spillover risk, in order to reduce the risk of human infection. It is informed by, among other actions, biosurveillance in domestic and wild animals, people and the environment, understanding pathogen infection dynamics, and implementing intervention activities.

**Table 1:** Framework for pandemic prevention, preparedness and response (revised from [19]).

Overall goal	**Pandemic prevention** Primary (upstream) prevention to reduce the *likelihood* of spillover events	**Pandemic preparedness and response** Secondary (downstream) prevention to reduce the *impacts* of events resulting from spillover
Definition	Prevention of pathogen spillover to humans (primary prevention/upstream or deep prevention)	Prevention of pathogen spread in humans (secondary or downstream prevention)
Stages of intervention	Before spillover from animals to humans occurs	After spillover from animals to humans occurred
Approach	Identifying sources of risk and addressing them based on the relevant stakeholder(s) using a One Health approach	Containment, including to avoid inter- and intraspecies transmission and spillback from people to other animal species
Focus of intervention	Direct or indirect spillover of pathogens from animals to humans	Preventing pathogens from spreading in human population
Actions	Integrated One Health surveillance to detect and monitor threats/inform risk assessment. Addressing drivers of disease emergence, including human behaviors and activities that increase risk (e.g., certain conditions and practices associated with climate change, land use change, wildlife trade, food systems). Development and implementation of risk reduction activities including biosecurity and vaccination for infection prevention to avoid animal–human or human–animal transmission	Early pathogen detection in humans, vaccines, improving health systems, health promotion, social and behavioral changes, drugs, risk reduction interventions (both human and animals), sanitary measures
Proposed focus of current Instruments	WHO pandemic instrument [3] One Health Joint Plan of Action [16] World Bank Pandemic fund [2] Nature for Health Biodiversity for Pandemic Prevention Multi-Partner Trust Fund [20]	
Relevant international agreements and tools	International agreements on wildlife trade [21], climate change [22], biodiversity [12], WOAH Performance of Veterinary Services (PVS) [23]	International Health regulations (IHR) [24], Joint External Evaluation (JEE) [25]

## Scope of prevention of spillover

An important principle is that spillover of pathogens from a natural source only occur after direct or indirect contact between the pathogen (e.g., via an infected host/environment) and people at interfaces between humans, animals, and the environment. Animals and biodiversity do not present an inherent risk per se; risk is created by human behavior that places humans and other species in risky contact that increase chances for spillover. Understanding the presence, diversity, evolution and characteristics, distribution, and infection dynamics of pathogens in the host using a One Health approach can assist in identifying risk factors for spillover, and hence opportunities/critical control points for spillover prevention, although a more generic approach can also be taken in the absence of thorough knowledge of these aspects. Depending on the context and existing evidence, trade-offs of possible interventions, and resource requirements, this can be complex and might require several interventions at different risk interfaces. However, it is possible and has been shown to be more cost-effective than relying on response activities [1,4,5]. Knowledge of the human–animal–environment interface and how this has changed over time (e.g., changes in land use, which species are hunted or farmed, farming methods, food systems, animal trade, infrastructure, and industry developments) are essential to inform approaches for prevention. It is therefore essential to also invest in research and the socioeconomic factors that change these. This also applies for vector-borne diseases, where knowledge on habitat suitability, climate factors, and host abundance can be used for risk assessment. Where available, information on presence, excretion, and pathogenicity of specific pathogens also can inform risk assessment. However, even without that information, knowledge of possible exposure routes across the human–animal–environment interface can be used to identify critical control points, and modification of human behaviors can be introduced to reduce human infection risk in a generic, multi-hazard fashion. Specific factors related to hunting, capturing, farming, and slaughter/preparation of wild animals; intensive/high density livestock farming especially linked to inadequate biosecurity; trade in live animals and animal products; deforestation, extractive industries, and encroachment into wildlife habitat; agricultural expansion and intensification; and urbanization and habitat fragmentation are often important in shaping risk. Overarching drivers, such as climate change, food security, basic animal and human health, animal welfare practices, poverty, and socioeconomic inequalities, should also be considered in the prevention of spillover.

### Concluding remarks

There are several ongoing discussions, revisions, and developments of new instruments, funding strategies, tools, and plans that can potentially play a role in the prevention of future pandemics, including the pandemic instrument [3] and Global Biodiversity Framework [11]. Still, prevention of spillover is not yet prioritized, and the drivers for zoonotic pathogen transmission to humans in the P-P-R triad are not specifically addressed. If there is to be serious commitment combined with good evidence, knowledge, attitude, and practices to reduce the risk of occurrence of future pandemics—versus just trying to reduce pandemic spread through improved responses—it is essential that discussions and actions on pandemic prevention focus on the primary prevention of pathogen spillover as the first decisive step. It is also critical that environmental initiatives, e.g., the Post-2020 Global Biodiversity Framework (GBF) [12], are implemented to explicitly include reduction of spillover risk and, consequently, the emergence of future pandemics as an objective. Encouragingly, Target 5 of the GBF does focus on spillover risk reduction, but achieving that outcome will require to link biodiversity-focused finance and action with the risk reduction expertise of human and animal health sectors. In order to ensure clarity of purpose and the ability to implement meaningful and equitable outcomes, a One Health approach should be emphasized as an overarching strategy [13,14]. The One Health High Level Expert panel (OHHLEP) and the Quadripartite are providing strategic direction on these aspects, specifically in the OHHLEP Theory of Change [15,16], and the Quadripartite global One Health Joint Plan of Action [16]. Addressing spillover risk also should consider specific geographic contexts and people’s socioeconomic and cultural backgrounds, while avoiding infringement of human rights, including those of indigenous communities, in line with the foundational One Health principles [14]. Spillover prevention should follow a One Health risk reduction approach, recognizing that many anthropogenic behaviors and activities result in environmental changes and socioeconomic factors that increase spillover risk—which can be mitigated with pragmatic, anthropogenic actions **(Box 2).** Preventing future pandemics will require sustainable investment in spillover prevention. Several opportunities are emerging such as the World Bank Pandemic fund for Pandemic P-P-R [17], Global Funding facility (GFF) [17], and Global Environmental facility (GEF) [18]—but require a strategy for alignment, filling gaps, and sustainment of risk reduction. As the world contemplates a global pandemic summit at the UN General Assembly this September, it is clear that pandemic prevention at the source cannot continue as an afterthought—a much larger commitment is overdue and sorely needed to prevent future pandemics.

**Box 2. Illustrative results framework for pandemic prevention at source**

Depending on the context and relevant sectors, there are different possible entry points and metrics for reducing the risk of spillover. Because of poor baseline data on spillover risk, particularly in settings where events may go undetected until developing into epidemics, precisely demonstrating the impact of changes in policies or practices on cases or events may be challenging, and may be further complicated by narrowing down spatial and temporal scales in which events leading to detection may be relatively rare. The use of intermediate indicators can inform on whether processes are being undertaken that help to make risk reduction more systematic. For example, for highly pathogenic avian influenza viruses, measuring only the number of human cases misses key opportunities for reducing risks and wider impacts of zoonotic influenza, such as enhancing biosecurity or utilizing zoning laws to restrict poultry farming near wetlands. The following illustrative indicators can be used as a starting point by countries, with greater precision of metrics based on the availability of data.

*Impact indicators*

- Reduced number of spillover events (e.g., as measured by number of disease outbreaks or incidence of disease index cases)
- Spillover risk reduction as a result of risk mapping and mitigation measures in place

*Intermediate indicators*

- Number of practices driving risk identified
- Number of actions taken to address practices driving risk
- Number of sectors/stakeholder groups engaged in spillover risk reduction efforts
- Amount of financial resources allocated to spillover risk reduction
- Spillover risk mapped and up to date
- Spillover interfaces (places and activities that put people and wildlife in close contact) identified at national or subnational levels
- Risk assessment(s) conducted and up to date for zoonotic pathogens at each specific spillover risk interface identified
- Spillover risks considered in land use and other development projects planning and impact assessment criteria

Source: authors.
